# Unusual Complications of Quinalphos Poisoning

**DOI:** 10.1155/2013/809174

**Published:** 2013-05-12

**Authors:** Stalin Viswanathan

**Affiliations:** Department of Medicine, Indira Gandhi Medical College, Pondicherry 605009, India

## Abstract

This 40-year-old man was treated for suicidal quinalphos 25%EC consumption. He developed intermediate syndrome with normal response to repetitive nerve stimulation, pancreatitis with high enzyme elevations, and normal computed tomography and excreted black, brown, and orange urine sequentially over the first nine days of hospitalization. The last complication has not been previously reported with any organophosphate compound. He finally succumbed to complication of ventilator associated pneumonia related septic shock and ventricular tachycardia.

## 1. Introduction

Organophosphate (OP) poisoning is a common entity in rural India. Intermediate syndrome is the major cause of mortality and morbidity in this poisoning. Urinary discoloration arising from OP poisoning has not previously been described. Pancreatitis has been reported with other OP compounds but not with quinalphos. We describe the fatal course of a 40-year-old man with quinalphos consumption who had developed black, red, and orange urine sequentially along with intermediate syndrome, pancreatitis, hypernatremia, and renal failure. Urinary discoloration and pancreatitis have never been reported as a complication of quinalphos poisoning and hence this report.

## 2. Case

**Figure 1:**
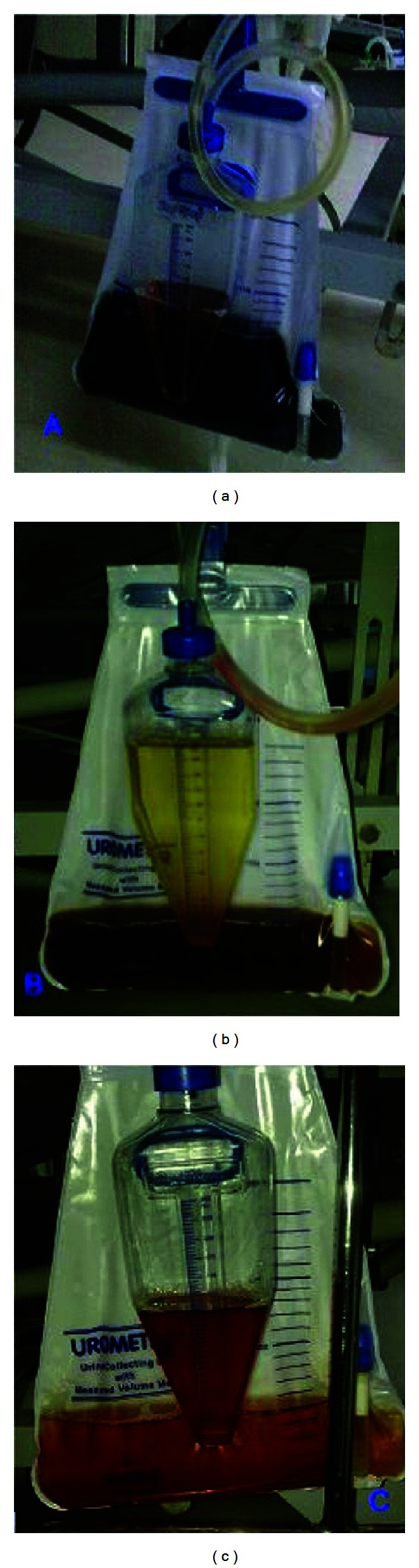
(a) Black urine in urine-bag seen on day three (b) Brown urine seen between fifth and eighth days (c) Orange urine seen on the 9th day.

This clerk was referred for respiratory distress five hours after consuming ~25 mL of quinalphos 25%EC. Examination at admission revealed pulse of 70 beats/min, normotension (100/70 mmHg), tachypnea (40/min), profuse diaphoresis, lacrimation, fasciculations, labored breathing, and right infrascapular crackles. His trachea was intubated immediately, and atropine infusion (5 mg/hour) was initiated along with ceftriaxone and metronidazole. Elevated plasma choline esterase levels, hypokalemia, and neutrophilic leukocytosis were seen (Table 1). Twenty-six hours later he developed neck flop, and within 48 h he had bilateral ophthalmoplegia and ptosis, limb weakness (power 3/5), and generalized areflexia. Magnesium levels and thyroid profile were ordered for hypokalemic weakness (Table 1). On the 3rd day, black urine was noted in the urine-bag (Figure 1(a)), while brown-red-orange color was noted between 5th and 8th days (Figures 1(b) and 1(c)). Urine examination on day 3 revealed pH 6.5, albumin 2+, hemoglobin positivity, and myoglobin negativity. Serum creatinine kinase (CK) was normal, while lactate dehydrogenase (LDH) and bilirubin were elevated (Table 1) and hematocrit had fallen to 36. On the fourth day, nerve conduction studies in the median, ulnar, and peroneal nerves revealed temporal dispersion but with normal distal latency, amplitude, and velocity. Repetitive nerve stimulation with 3 Hz and 10 Hz revealed no decremental response, while the response was equivocal with 20 Hz.

Serum sodium began to rise from day six (156 mEq/L) and persisted until the 15th day (163 mEq/L), while K^+^ remained low to normal (3.1 to 4.3 mEq/L). Urinary sodium and potassium were 17 mEq/L and 25 mEq/L, respectively. On day seven, patient had tachycardia and vomiting of nasogastric feeds for which pancreatitis was suspected. Amylase and lipase levels were high (Table 1): aggressive fluid correction and total parenteral nutrition were then instituted. Abdominal computed tomography (CT) was normal. Klebsiella pneumoniae was cultured in his endotracheal aspirate on 9th day and cefepime was administered. On the same day his urine began to change to orange. From day 11 onwards, he continued to have low grade fever. Ultrasonogram abdomen was normal except for minimal interbowel fluid. Blood cultures grew pseudomonas—prulifloxacin and meropenem were added to his treatment and his fever reduced but the course was complicated by worsening hypotension and increasing drowsiness. Renal and liver dysfunction began on the 16th day and two days later, he succumbed to refractory shock, metabolic acidosis, and multiple-organ dysfunction. 

## 3. Discussion

Pesticide poisoning causes about a quarter of a million deaths every year [1]. Organophosphates (OPs) inhibit cholinesterase (AChE) causing excessive acetylcholine accumulation at the synapse with subsequent nicotinic hyperstimulation. Acetylcholine esterase inhibition greater than 80% causes failure of neuromuscular transmission. A normal response, a decrement-increment pattern, a severe decrement pattern, a progressive decrement pattern, or a combination of decrement-increment and repetitive fade are seen in patients with intermediate syndrome (IMS) on repetitive nerve stimulation (RNS) [1, 2]. Our patient had IMS with a normal response to RNS. Respiratory failure is generally associated with a progressive decrement pattern, but this was not the case in our patient. IMS has been associated with compounds such as fenthion, dimethoate, monocrotophos, parathion, combined ethyl-parathion and methyl-parathion, methamidophos, dichlorvos, and lack of oxime therapy [1]. 

Pancreatic involvement may range from enzyme elevations to necrotizing pancreatitis that necessitates surgery [3]. Pancreatitis in OP poisoning is generally benign although necrosis, pseudocyst, gut infarction, and uncontrollable hemorrhage have been reported [4]. Organophosphates cause excessive pancreatic cholinergic stimulation in the pancreatic acini and also cause ductular hypertension, interstitial edema and enzyme elevation [4–6]. Prevalence of acute pancreatitis has ranged from 0.5% to 62.5% [4, 5] and mortality rates have varied from 5 to 10%. Pancreatic enzymes were performed for unresolved tachycardia, vomiting, and hemoconcentration in spite of adequate fluid replacement and negative screen for infections and cardiac dysfunction. Elevation in transaminases, LDH, total counts, and drop in hematocrit were also observed, to suggest pancreatitis.

Quinalphos has not been previously reported to cause pancreatitis. Compounds that have been described as an etiology of pancreatitis include malathion, parathion, fenthion, coumaphos, diazinon, difonate, dimethoate, methomyl, meprobamate, carbofuran, dichlorvos, mevinphos, and propoxyfur [6, 7]. Studies in rats have shown that early and high dose atropine reduces incidence of pancreatitis by decreasing ductular pressure and also reducing histopathological changes in the pancreas [6]. Hyperamylasemia and severity of poisoning do not always correlate with pancreatitis in OPC poisoning [7], as in our patient where CT findings were surprisingly normal considering the enzyme elevations. A bedside ultrasonogram three days after CT, for evaluating fever, also showed a normal pancreas.

Black urine has been described since time of Hippocrates and has always been considered a poor prognostic sign in medical illness. Brownish-black or tea colored urine is generally seen in hemoglobinuria and myoglobinuria arising from sepsis, crush injuries, hypokalemia, and inflammatory myopathies and urine generally turns this color in presence of acidic urine [8]. It can also be associated with copper, phenol, cresol, alkaptonuria, melanuria, and treatment with L-dopa and alpha methyldopa [8]. Poisoning due to cresol [9], endosulfan, and paraphenylene diamine is known to cause black urine due to the presence of phenolic metabolites. Red urine is generally due to hematuria, but vegetables like rhubarb, beetroot (due to betalain and seen in 10 to 14% of population), and blackberries [8]; metabolites like methemoglobin, melanin, porphyrin, and homogenitisic acid; medications like chloroquine, deferoxamine, ibuprofen, iron sorbitol, nitrofurantoin, phenytoin, rifampicin, vitamin B12, doxorubicin, phenazopyridine, phenolphthalein, and topical application of azosulfamide (alternative/complementary medicine) can also cause a similar discoloration [8]. Orange urine has been attributed to hemoglobin, myoglobin, porphyria, methemoglobinemia, iron (hemochromatosis), beetroot pigment, rifampicin and conjugated hyperbilirubinemia [8, 10]. 

The only common thread among all the three colors is the urinary finding of heme. Hemolysis due to percutaneous exposure of trichlorfon has been reported in three instances [11]. It is possible that hemolysis due to the OP compound and hypokalemia has contributed to the black urine. Decreasing severity of hemolysis has probably contributed to the change in urine color from black to brown and subsequently to orange. Though cefipime initiation and orange urine occurred on the same day, cefepime was probably not causative since urinary heme was still positive. A catheter-related urinary infection was suspected, but cultures were sterile. Urinary color returned to normal on day 15 with urinary heme being negative. But phenolic compounds in the OP (or carbamate) and its metabolites can itself cause urinary discoloration [12]. Quinalphos (O,O-Diethyl O-2-quinoxalinyl phosphorothioate) is an organothiophosphate compound primarily metabolized by desertification to quinoxalin-2-ol. About 87% of quinoxalin-2-ol is excreted in urine and the remaining in bile. The phosphorothioate moiety is excreted as diethyl phosphate and diethyl phosphorothioate [13]. Hence a combination of phenolic metabolites, hemolysis, and renal failure contributed to urine discoloration in our patient. Hypernatremia has been attributed to the hypovolemia (hemolysis related), renal failure and hemoconcentration that was aggravated by pancreatitis. Renal failure and hepatitis occurred preterminally. The patient died due to complications—ventilator associated pneumonia and ventricular tachycardia.

In conclusion our patient had two unusual complications of organophosphate poisoning. Discolored urine in OP poisoning, possibly hemolysis and phenolic metabolites related, has not been described in medical literature. The second, pancreatitis has been reported with OP poisoning, but not with quinalphos. Emergency physicians and internists should watch carefully for developing subclinical pancreatitis by way of unexplained tachycardia, falling hematocrit, vomiting unrelated to cholinergic symptoms, and development of ileus and/or increasing abdominal girth. Also discoloration of urine in such patients may give clues to developing hemolysis, rhabdomyolysis, hydration status, and sepsis. 

## Figures and Tables

**Table 1:** Lab investigations of patient.

Investigation	Value	Normal values
Choline esterase (day 1)	412	3714–11513 U/L
Potassium (day 1)	3.1	3.5–4.5 mmol/L
Hematocrit (day 1)	48	40–48
Total WBC counts (day 1)	167	40–95 × 10^6^/L
Neutrophils (day 1)	84	40–60%
Magnesium	0.85	0.62–0.95 mmol/L
Sodium (day 6)	156	135–145 mmol/L
Thyroid stimulating hormone (day 2)	0.02	0.4–4.5 mIU/L
Free T3	3.19	3.7–6.5 pmol
Free T4	27.5	10.3–21.9 pmol/L
Creatine kinase	180	40–250 U/L
Lactate dehydrogenase (day 3)	825	110–200 U/L
Total bilirubin (day 3)	54	5–25 *μ*mol/L
Indirect bilirubin (day 3)	46	3.4–15.2 *μ*mol/L
Lipase (day 7)	685	0.51–0.73 *μ*kat/L
Amylase (day 7)	900	<250 U/L
